# Expression, Functional Characterization, and Solid-State NMR Investigation of the G Protein-Coupled GHS Receptor in Bilayer Membranes

**DOI:** 10.1038/srep46128

**Published:** 2017-04-07

**Authors:** Stefanie Schrottke, Anette Kaiser, Gerrit Vortmeier, Sylvia Els-Heindl, Dennis Worm, Mathias Bosse, Peter Schmidt, Holger A. Scheidt, Annette G. Beck-Sickinger, Daniel Huster

**Affiliations:** 1 Institute of Medical Physics and Biophysics, University of Leipzig, Härtelstr. 16-18, D-04107 Leipzig, Germany; 2 Institute of Biochemistry, University of Leipzig, Brüderstr. 34, D-04103 Leipzig, Germany

## Abstract

The expression, functional reconstitution and first NMR characterization of the human growth hormone secretagogue (GHS) receptor reconstituted into either DMPC or POPC membranes is described. The receptor was expressed in *E. coli*. refolded, and reconstituted into bilayer membranes. The molecule was characterized by ^15^N and ^13^C solid-state NMR spectroscopy in the absence and in the presence of its natural agonist ghrelin or an inverse agonist. Static ^15^N NMR spectra of the uniformly labeled receptor are indicative of axially symmetric rotational diffusion of the G protein-coupled receptor in the membrane. In addition, about 25% of the ^15^N sites undergo large amplitude motions giving rise to very narrow spectral components. For an initial quantitative assessment of the receptor mobility, ^1^H-^13^C dipolar coupling values, which are scaled by molecular motions, were determined quantitatively. From these values, average order parameters, reporting the motional amplitudes of the individual receptor segments can be derived. Average backbone order parameters were determined with values between 0.56 and 0.69, corresponding to average motional amplitudes of 40–50° of these segments. Differences between the receptor dynamics in DMPC or POPC membranes were within experimental error. Furthermore, agonist or inverse agonist binding only insignificantly influenced the average molecular dynamics of the receptor.

G protein-coupled receptors (GPCRs) belong to the largest class of mammalian membrane proteins and have received significant attention due to their high pharmacological relevance. In spite of extensive attempts to crystallize GPCRs, a successful strategy was only developed about a decade ago. Crystallization succeeded after intracellular loop 3 that was found to be most responsible for helix movement, was rigidified by introduction of T4 lysozyme, truncations, thermostabilization, or by anti- or nanobody binding^1^,^2^,^3^. Such receptor engineering proved to be a very successful strategy and led to a number of crystal structures solved for various GPCRs^4^. But proteins are dynamic molecules that do not possess a unique and rigid conformation, but are best described as dynamic ensembles of more or less different structures^5^,^6^. This particularly applies to membrane proteins, which reside in liquid-crystalline bilayers that are also highly dynamic^7^.

In spite of the success of crystallography, the need to study native GPCRs in their relevant membrane environment remains as GPCRs are highly dynamic molecules that undergo various conformational changes upon activation by an extracellular ligand and subsequent G-protein interaction^8^. At this point, GPCR dynamics have to be addressed explicitly. The most important dynamic structural alteration has been observed for helix 6 of the heptahelical membrane proteins. Specific labeling of the protein with fluorescence^9^,^10^, EPR^11^,^12^, or NMR^13^,^14^,^15^,^16^,^17^,^18^,^19^ probes have highlighted the dynamical alterations of the molecule upon inverse agonist or agonist binding as well as G-protein interaction. The most complete set of data that describes the interesting structural dynamics of GPCRs is available for the β_2_-adrenergic receptor (β_2_AR). Unliganded receptor and inverse agonist bound β_2_AR seem to exist in two inactive conformations that exchange rapidly with a frequency in the kilohertz range^18^. Agonist binding shifts the equilibrium towards a conformation that can bind cytoplasmatic G-protein, this process is incomplete resulting in an even increased conformational heterogeneity, which includes the active, possibly several intermediate, and the inactive states. Only G-protein binding results in the complete transition to the active conformation, which essentially abolishes the aforementioned dynamics^18^. This and other data suggests that the assumption of just one active receptor structure may represent a too simplistic representation of the function of GPCRs^9^.

Energy landscapes are a useful tool to integrate GPCR structure, dynamics, and function^8^,^20^ and represent a general means to describe the dynamic character of proteins^6^. For GPCRs, models of the energy landscapes of the molecules upon activation and G-protein binding show pronounced minima for the ground state and the active state, locked by the G-protein^21^. However, there are also several local minima, separated by low energy barriers that are populated upon binding of agonists and inverse agonists as demonstrated for the β_2_AR^18^.

So far, explicit data on the molecular dynamics of GPCRs is rather limited. In addition to the aforementioned data on the dynamic activation of GPCRs, one study has addressed the molecular dynamics of the CXCR1 receptor reconstituted in bilayer membranes^22^. This investigation came to the conclusion that the receptor undergoes axially symmetric rotational diffusion in the membrane and about 10% of the residues are subject to larger amplitude motions. In contrast, the molecular dynamics of the human neuropeptide Y receptor type 2 has been systematically studied revealing that the individual segments of the molecule are subject to large amplitude motions, expressed by average order parameters between 0.55 and 0.67 in the protein backbone, which corresponds to amplitudes of the segmental motions up to about 50° 23,24. Here, we report results on the molecular dynamics of the human growth hormone secretagogue (GHS) receptor, which plays a key role in the regulation of appetite and food intake and, therefore, represents an interesting target for intervening with eating disorders^25^. The ligand of the GHS receptor is the octanoylated growth hormone ghrelin consisting of 28 amino acids^26^,^27^,^28^. We used solid-state NMR spectroscopy to study the molecular dynamics of the GHS receptor reconstituted into DMPC or POPC membranes.

## Results

### Pharmacological characterization of the recombinant GHS receptor

**Figure 1:**
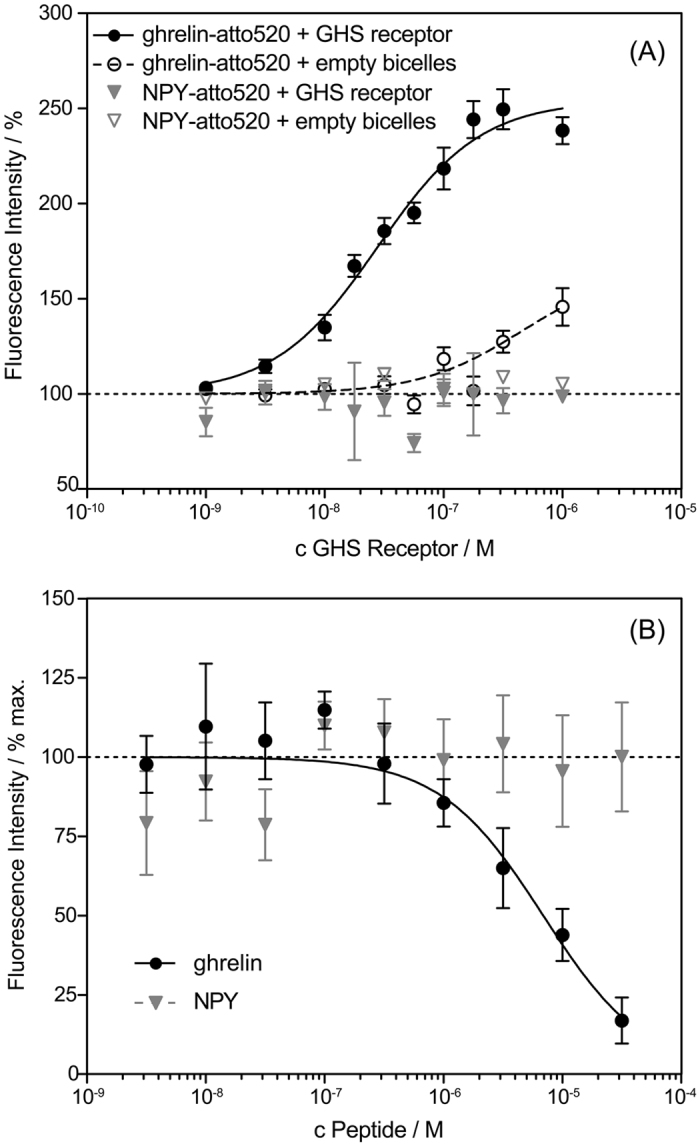
(**A**) Saturation binding of atto520-labeled ghrelin (c = 100 nM) to increasing amounts of GHS receptor-containing bicelles or empty bicelles. As a control, atto520-labeled NPY was used, which did not display enhanced fluorescence in the presence of GHS receptor-loaded or empty bicelles. Data reflect fluorescence enhancement upon binding. The inflection point (EC_50_ = 28 nM) for GHS receptor binding is approximately at the limit of the assay of L_total_/2 = 50 nM; demonstrating high functionality of the system. (**B**) Displacement of atto520-ghrelin binding to the GHS receptor by unlabeled ghrelin. In contrast, neuropeptide Y (NPY) was not able to displace the bound atto520-ghrelin ligand. Results represent mean +/− SEM of three independent assays each performed in triplicates.

Prior to biophysical investigation of the GHS receptor, a detailed pharmacological characterization was carried out using homogenous fluorescence assays as described in the literature^29^. Figure 1A shows the dose-dependent atto520-ghrelin ligand binding plots of the GHS receptor.

The inflection point for GHS receptor binding was determined at EC_50_ = 28 nM, which is approximately at the limit of the assay; demonstrating high functionality of the system. The assay limit is dictated by the total concentration of the labeled ligand (L_total_). Fluorescence enhancement will be maximal when all labeled ligand molecules are bound by receptor. Assuming a 1:1 stoichiometry, half-maximal signal is reached at receptor concentrations equivalent to L_total_/2 + K_d_^30^, leading to an assay limit of L_total_/2 for high-affinity systems. Unfortunately, the use of lower tracer concentrations closer to the reported sub-nanomolar K_d_ was obviated by the sensitivity of the fluorescence reader. Ghrelin also binds to empty phosphatidylcholine bicelles, but at much higher concentration as determined before^26^. As an additional control, we used fluorescently labeled neuropeptide Y (atto520-NPY), which should neither bind to the GHS receptor nor to empty phosphatidylcholine bicelles. Accordingly, no change in fluorescence was recorded.

Next, displacement experiments were carried out at a tracer peptide concentration far above the K_d_, resulting in a large Cheng-Prusoff shift and further distortion by ligand depletion^31^,^32^. Applying these correction factors and using the K_d_ of 0.15 nM as reported by Els *et al*.^33^, a K_i_ of 10 nM can be calculated from the IC_50_ of 6870 nM. This corresponds well to values observed previously both *in vivo* and *in vitro*^34^,^35^. The specificity of displacement was further underlined by using NPY as a control, which did not result in displacement of the atto520-ghrelin ligand (Fig. 1B).

### Static ^15^N CP spectra of the GHS receptor in DMPC

A standard method to characterize membrane proteins is the analysis of static ^15^N CP NMR spectra of uniformly ^15^N labeled membrane protein^36^. Fully rigid membrane proteins that do not undergo axially symmetric rotational diffusion show NMR spectra, which are characterized by broad anisotropic lineshapes with an asymmetry parameter η ≠ 0. Axially symmetric rotational diffusion of the membrane protein is manifested by anisotropic lineshapes indicative of axially symmetric chemical shift anisotropy tensors (η = 0). Superimposed to these broad lineshapes, large amplitude motions give rise to very narrow lines detected at the isotropic chemical shift of the respective resonances^22^,^24^. However, as static ^15^N NMR spectra are almost always acquired using cross polarization (CP), the recorded spectra show a strong bias towards rigid structures and dynamic features are only detected if sufficiently long CP contact times are used^24^.

**Figure 2:**
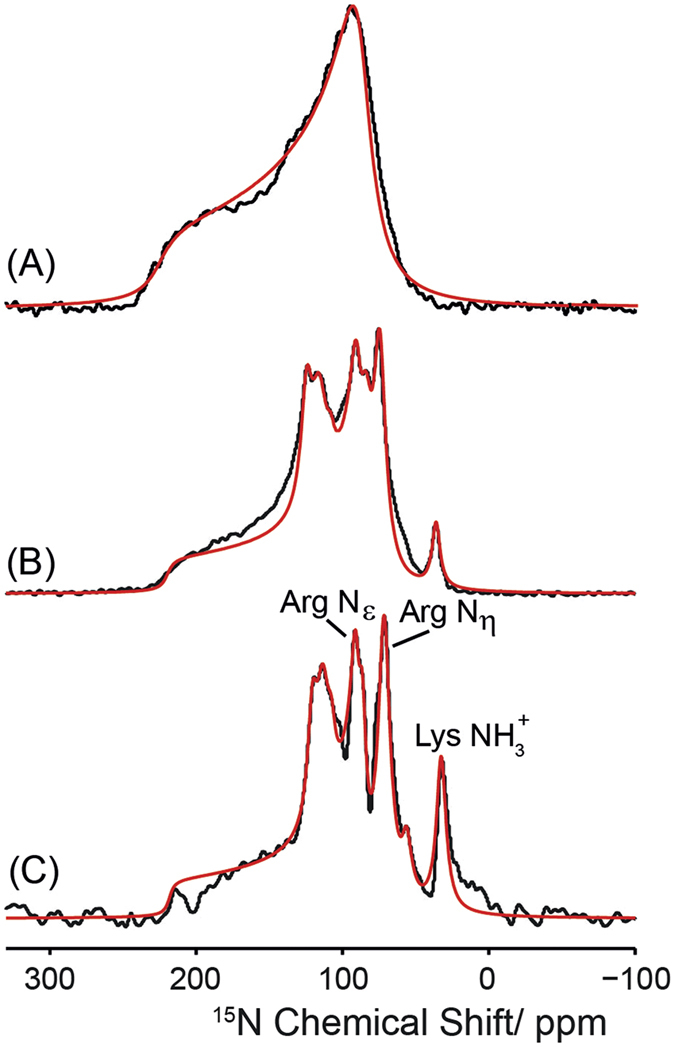
Static ^15^N CP NMR spectra of the uniformly ^15^N-labeled GHS receptor reconstituted into DMPC membranes at a temperature of 37 °C. NMR spectra were acquired using CP with contact times of 70 μs (**A**), 2 ms (**B**), and 8 ms (**C**). Assignment of the prominent side chains is given in the NMR spectrum acquired at the longest CP contact time. Superimposed with the experimental data, best fit numerical simulations of the spectral line shapes are shown.

Figure 2 shows static ^15^N NMR spectra of the GHS receptor reconstituted into DMPC membranes recorded with CP contact times of 70 μs (A), 2 ms (B), and 8 ms (C) at a temperature of 37 °C. All spectra show anisotropic lineshapes that can be simulated using an axially symmetric CSA tensor with a span of Δσ = 128–138 ppm. This is an indication that the receptor is in a monomeric form. As longer CP contact times are used, also narrow ^15^N NMR lines are detected, which are indicative of receptor segments undergoing large amplitude motions. These can be assigned to the Lys and Arg side chains resonating at higher magnetic field, but also to amide signals of the receptor backbone at chemical shifts of ~108, 116, and 124 ppm. This observation indicates that in addition to the axially symmetric rotational diffusion of the GHS receptor in the membrane, significant portions of the molecule also undergo large amplitude motions. We have previously quantified the proportion of highly mobile segments of the Y_2_ receptor by acquiring static ^15^N NMR spectra as a function of CP contact time as well as directly excited ^15^N NMR spectra^24^. It could be shown that static ^15^N CP spectra recorded at a CP contact time of 8 ms can be readily quantified to provide the relative proportion of relatively rigid and highly mobile residues^24^. Following this analysis, we can estimate that about 25% of the residues of the GHS receptor undergo large amplitude motions in DMPC membranes, which is significantly smaller than what has been determined for the Y_2_ receptor, for which about 40% of the entire spectral intensity of the static ^15^N NMR spectra was attributed to the isotropic signals.

### ^13^C MAS NMR studies of the GHS receptor in membranes

^13^C NMR experiments recorded under MAS conditions provide a more direct way to investigate the molecular dynamics of membrane proteins. Provided that high resolution can be achieved either by selective labeling^37^ or quasi-crystalline preparation of the membrane protein of interest^38^,^39^,^40^, even side-specific dynamics information such as segmental order parameters can be determined. In the case of G protein-coupled receptors, spectral resolution is limited and only a single case has been reported in the literature where a large amount of sequential assignments have been achieved^41^. In our hands, the GHS receptor as well as the Y_2_ receptor reconstituted into DMPC or POPC membranes^23^,^24^,^42^ yielded relatively broad NMR lines under MAS at moderate frequencies rendering sequential assignments impossible so far.

**Figure 3:**
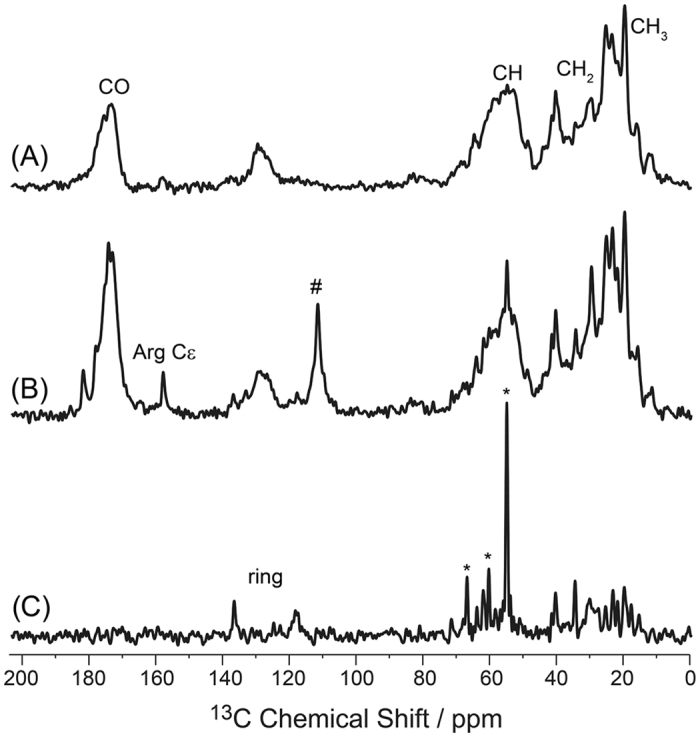
^13^C MAS NMR spectra of the uniformly ^13^C-labeled GHS receptor reconstituted into chain perdeuterated DMPC-*d*_54_ membranes using different ^13^C excitation pulse sequences. (**A**) ^13^C CPMAS spectrum using a CP contact time of 20 μs, (**B**) directly excited ^13^C MAS NMR spectrum. (**C**) ^13^C INEPT NMR spectrum. All NMR spectra were recorded at 37 °C at a MAS frequency of 7 kHz using Spinal64 decoupling with a radio frequency amplitude of ~65 kHz. The asterisk denotes lipid signals from the DMPC headgroup and glycerol backbone and the hashtag indicates the Teflon signal from the probe that is detected in directly excited ^13^C MAS NMR spectra.

Figure 3 shows typical ^13^C MAS NMR spectra of the uniformly ^13^C labeled GHS receptor reconstituted into DMPC-*d*_54_ membranes with perdeuterated lipid chains. In both ^13^C CPMAS (A) and directly excited ^13^C MAS NMR spectra (B), the NMR lines of the receptor are relatively broad and dispersed in a narrow chemical shift region as typical for α-helical membrane proteins^41^. Directly excited ^13^C MAS NMR spectra (B) show several narrow NMR lines, indicative of highly mobile sites especially for the ^13^CO resonances and the aromatic rings. A narrow signal from the Arg ^13^Cε side chain can also be well resolved. Some narrow lines can also be observed in the ^13^C INEPT spectrum of the GHS receptor in particular in the side chain region (C). However, the INEPT spectrum in the backbone region is dominated by lipid signals, which means that the fraction of receptor segments that undergo large amplitude motions is relatively small. Again, this is somewhat in contrast to our previous investigations on the molecular dynamics of the Y_2_ receptor^23^, where ^13^C INEPT spectra showed a larger proportion of narrow lines. Qualitatively, the ^13^C MAS NMR spectra of the GHS receptor reconstituted into POPC membranes are similar (data not shown), however, due to the lack of deuteration, more lipid signals are detected in the directly excited and INEPT NMR spectra.

### Motional amplitudes of the GHS receptor in POPC and DMPC membranes

For a more quantitative discussion of the dynamics of the GHS receptor, we carried out ^1^H-^13^C dipolar coupling measurements using the DipShift pulse sequence^43^, which is a separated local field experiment^44^. Molecular motions of the C-H bond vector with a given amplitude scale down the dipolar couplings resulting in a reduced coupling constant provided these motions are faster than ~40 μs. Typical dipolar dephasing curves for two independent preparations and measurements are shown in Fig. S1. From this motional averaging, a molecular order parameter can be derived, which describes the amplitude of the motions of the respective segment. Typically, dipolar dephasing curves obtained under MAS conditions are measured, from which the exact value for the dipolar coupling is extracted in a simulation. All preparations and measurements were done at least twice; error bars represent the difference between two independent preparations.

**Figure 4:**
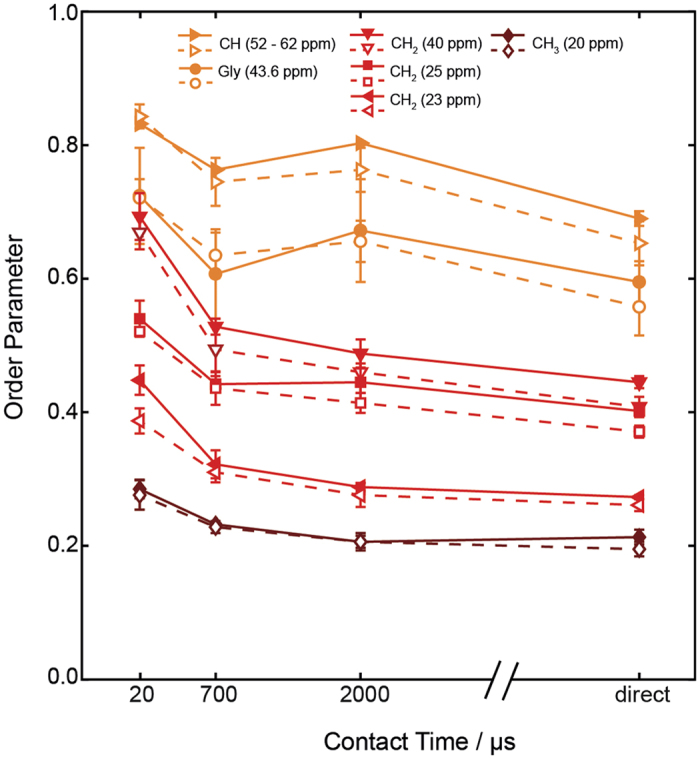
^1^H-^13^C NMR order parameters for backbone and side-chain carbons of the uniformly ^13^C labeled GHS receptor reconstituted into POPC (filled symbols) and DMPC membranes (open symbols) at varying CP contact times and a temperature of 37 °C. C-H dipolar coupling constants were measured using DipShift experiments and averaged over several signals in the same ^13^C chemical shift region or for specific peaks.

The average order parameters of the resolved segments of the GHS receptor in POPC and DMPC membranes are reported in Fig. 4. Similar to the static ^15^N measurements, ^1^H-^13^C DipShift experiments that use a cross polarization transfer step are biased by the presence of motions in the molecule. At short CP contact time, only the rigid segments are efficiently excited and contribute to the spectral intensity, which most likely involve the transmembrane helices of the receptor. DipShift experiments acquired at longer CP contact times also excite the more mobile parts of the receptor, which are constituted by larger loops and tail regions. All ^13^C sites are equally excited if directly excited DipShift experiments are carried out. As a consequence, the measured order parameters are largest for short CP contact times and decrease upon increase of the contact times. Lowest order parameters are always determined for the directly excited DipShift experiments. In addition to the contact time dependence of the order parameters, we also observe order parameter differences for the individual positions in the protein backbone and sidechain. Highest order parameters are observed for the Cα and Gly Cα sites, followed by CH_2_ groups in the side chain and lowest order parameters are observed for the CH_3_ groups. Order parameters of the GHS receptor in POPC membranes are typically slightly higher than those in DMPC bilayers but the difference is much smaller as determined for the Y_2_ receptor before^23^,^24^. Without the motional bias of the CP experiment, lowest order parameters determined for the protein backbone were 0.60–0.69 for POPC and 0.56–0.65 for DMPC membranes. These values are slightly higher than what has been observed for the Y_2_ receptor before^23^,^24^. Order parameters values of the GHS receptor in DMPC and POPC membranes are reported in Tables S1 and S2 in the Supporting Information.

As INEPT-based polarization transfer also provided some receptor signals, we measured the motionally averaged dipolar couplings of receptor segments that could be excited by INEPT using the separated local field experiment r-PDLF^45^. Residues that can be detected by INEPT-based pulse sequences have to be highly mobile, which is the case especially for the termini of the receptor^22^,^41^. Figure S2 shows experimental r-PDLF spectra of the GHS receptor in the presence of its ligand ghrelin as well as numerical simulations to determine the motionally averaged dipolar coupling for the signals detected at 23 and 40 ppm, respectively. Such chemical shifts are typical for the aliphatic side chains and could correspond to Cγ sites of amino acids such as Lys, Thr, or Val and the Cβ site of Leu, respectively. The determined order parameters are 0.09 for the peak at 23 ppm and 0.05 for the peak at 40 ppm.

### Order parameters of the GHS receptor in the presence of the agonist and an inverse agonist

**Figure 5:**
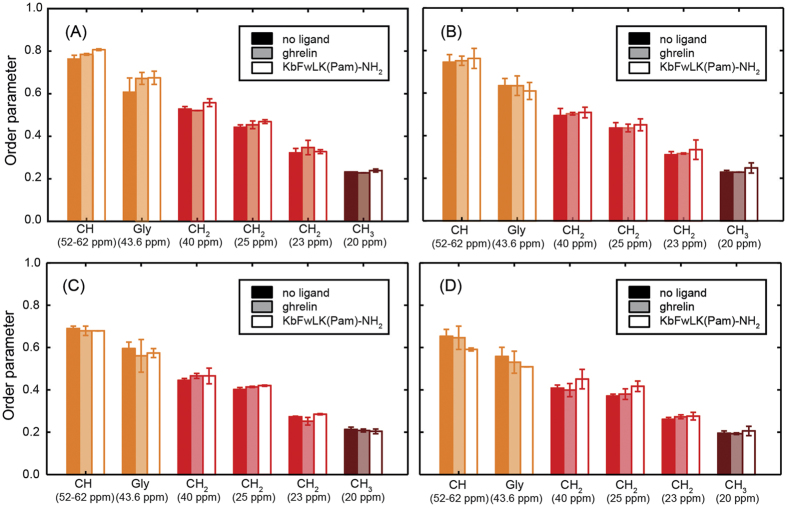
Summary of the order parameter measurements of the GHS receptor in the absence of ligand (filled bars) and in the presence of the agonist ghrelin (light filled bars) and the inverse agonist KbFwLK(Pam)-NH_2_ (empty bars) performed this study. Order parameters were determined from DipShift experiments conducted using CP excitation at a contact time of 700 μs (**A**,**B**) as well as by direct excitation (**C**,**D**). The GHS receptor was reconstituted into POPC (**A**,**C**) or DMPC membranes (**B**,**D**) and investigated at a temperature of 37 °C.

Agonists and inverse agonists shift the equilibrium of active and inactive G protein-coupled receptor towards the active or inactive conformation, respectively. As the GHS receptor has been reported to display a ~50% constitutive activity^35^,^46^, studying the molecular dynamics of the receptor in the presence of either agonist or inverse agonist represents an interesting topic^8^. Figure 5 shows the order parameters of the GHS receptor in the absence as well as in the presence of either the agonist ghrelin or the inverse agonist KbFwLK(Pam)-NH_2_ recorded in DipShift experiments that were excited by CP at a contact time of 700 μs (A, B) or by direct ^13^C excitation (C, D) both in POPC (A, C) and DMPC membranes (B, D). Order parameters determined by CP at a contact time of 700 μs are generally higher than those obtained from directly excited DipShift experiments irrespective of the presence or absence of any ligand. Order parameters determined from the CP based DipShift experiments at 700 μs contact time, which preferentially detects the more rigid structures show a tendency to be slightly higher for the ligand bound GHS receptor. However, this is just a trend, which is not consistent for all receptor segments studied and only in a few cases outside of the error margin of the experiment, which was determined from preparations and measurements carried out in duplicate. Order parameters determined by direct ^13^C excitation, which do not have a bias by molecular motions with varying amplitudes are identical within the experimental error for all three studied cases. No systematic differences between receptor preparations in saturated DMPC versus monounsaturated POPC membranes were observed. All order parameters are reported in Tables S1 and S2.

## Discussion

We describe the expression, functional reconstitution in lipid membranes, and preliminary NMR investigation of the human GHS receptor from *E. coli* culture. The receptor could be labeled with stable ^13^C and ^15^N isotopes allowing for an initial characterization by solid-state NMR. At the current preliminary state, unfortunately, the spectral resolution does not allow for a site-specific assignment of the NMR lines and the parameters that have been determined from the solid-state NMR measurements are averaged over the entire molecule. This situation lags behind the capabilities of modern NMR spectroscopy as demonstrated for other GPCRs^41^ or membrane proteins of similar size^38^,^47^. We suspect that the mobility of the molecule in fluid membranes is the reason for the limited spectral resolution. For a more site-specific approach, expression in auxotrophic *E. coli* strains or cell free expression might improve the situation. Although with the current initial results, only information averaged over the entire molecule can be obtained, there are some important conclusion that can be drawn with regard to the biological meaning of the results of this study.

### The explicit studies on the GHR receptor dynamics confirm that GPCRs are highly dynamic membrane proteins

Our data of the ground state dynamics of the GHS receptor in membranes indicate segmental fluctuations and rapid conformational transitions of the molecule on a submicrosecond timescale. This correlation time window includes local dynamics, mobility of secondary structure elements as well as more collective motions. In addition to the axially symmetric rotational diffusion of the protein in the membrane, we detected fluctuations in the backbone of the receptor described by relatively low order parameters. Although no site resolution has been achieved, the data allow some conclusions about domain-specific dynamics. At the very short CP contact time of 20 μs, only the rigid segments of the receptor such as the central parts of the transmembrane α-helices are excited, these segments show relatively low conformational freedom expressed by a high order parameter of 0.72–0.84. However, this is clearly smaller than what has been measured for the transmembrane segments of *Anabaena* sensory rhodopsin, where site-specific order parameters of 0.9 and higher have been reported for the transmembrane helices (*vide infra*)^38^. In contrast, we can also excite very mobile receptor domains using the INEPT polarization transfer scheme, which revealed that signals with order parameters lower than 0.1 can be detected, which likely belong to the highly mobile termini. This domain-specific data demonstrates that the conformational flexibility of GPCRs is broadly distributed between the individual segments from highly mobile to very restricted.

These dynamic properties represent a consequence of the energy landscapes, on which these molecules exist^20^, which is described as a rugged surface that allows for a multitude of conformational states, separated by low energy barriers on the order of the thermal energy that can be overcome by fluctuations of various amplitudes^6^. This translates into an inherent flexibility of the individual segments of the GPCR at equilibrium. Such fluctuations have also been observed in recent molecular dynamics simulations of the neurotensin receptor^48^. This receptor shows quite significant root mean square fluctuations in the backbone in particular for the loop and tail structures. Interestingly, these molecular dynamics simulations show that thermostabilization of the neurotensin receptor leads to a significant reduction of these fluctuations^48^. Also, crystallographic B factors of this and other GPCRs are relatively high and crystal structures often lack electron density for loop and tail structures, which suggests that these segments may undergo large amplitude motions or are statically disordered^11^,^49^.

### The GHS receptor is more mobile than other membrane proteins of comparable size

Compared to other membrane proteins of similar size^38^,^50^,^51^, the order parameters of the GHS receptor are significantly lower. For the backbone, the measured order parameters translate into average fluctuations with amplitudes on the order of 40–50°. Compared to the neuropeptide Y_2_ receptor using the same set of experiments^23^,^24^ the GHS receptor shows slightly higher order parameters. An obvious difference between the GHS and the Y_2_ receptor is the distribution between the lengths of the loops and tails on the one and transmembrane α-helical segments on the other hand. In the GHS receptor, about 56% of the residues constitute loops and tails, while this number increases to 61% for the Y_2_ receptor. In particular, the termini of the Y_2_ receptor are significantly longer (in total 107 vs. 80 residues). Assuming that loops and termini are more flexible than the helical segments, the difference in the dynamics between the two molecules can be well accounted for.

It is obvious that β-barrel membrane proteins are intrinsically more stabilized than molecules of predominantly α-helical structure. Intrastrand hydrogen bonds lead to high structural homogeneity of β-barrels, which are expressed in high backbone order parameters on the order of 0.9 as observed for OmpA from different sources^47^,^51^. On the secondary structure level, isolated α-helices are more flexible. In particular, the helix ends are subject to dynamic processes that have been seen both experimentally^52^ and in molecular dynamics simulations^53^. Furthermore, transmembrane α-helices are also known to show some interesting structural plasticity, which includes dynamic transitions between α-helical and β-sheet structures^54^,^55^. The α-helical protein colicin Ia in the membrane bound state showed order parameters between 0.88 and 0.93 for the backbone at a CP contact time of 0.7 ms^50^, which is much higher than what has been observed for the GHS receptor under these conditions here (order parameters for the backbone at a CP contact time of 0.7 ms were between 0.61 and 0.81).

### The general heptahelical architecture of a membrane protein is not the determining factor for its molecular dynamics in fluid lipid membranes

GPCRs appear to be specifically more flexible than heptahelical microbial rhodopsins such as bacteriorhodopsin^56^,^57^, proteorhodopsin^39^, or *Anabaena* sensory rhodopsin^38^, for which more ordered structures with higher order parameters have been reported. This may in part be due to the 2D crystalline nature of the host membrane, but also membrane reconstituted microbial rhodopsins in the lamellar liquid-crystalline lipid phase showed high order^39^. Although Gly-rich motifs (GxxG and GxxxG) are discussed to be responsible for structural plasticity of fusion peptides^58^ the transmembrane regions of the GHS receptor have fewer Gly residues than the respective segments of both proteorhodopsin or *Anabaena* sensory rhodopsin. Therefore, the only obvious difference in the topology of the GPCRs and the microbial rhodopsins are the significantly longer loops that connect the transmembrane segments, which likely provides the molecular origin for the enhanced mobility of the interesting GPCRs.

### Agonist or inverse agonist binding does not alter the average dynamics of the GHS receptor

Another obvious difference between individual members of the GPCR family is the constitutive activity of the GHS receptor, which is on the order of 50%^35^,^46^. As well investigated for the β_2_ adrenergic receptor, ligand binding alters the shape of the energy landscape and the receptor populates different conformations^8^,^20^. However, agonist bound β_2_ adrenergic receptor was found to be also highly dynamic and to interconvert between inactive, intermediate, and active conformations^18^. To further investigate the dynamics of the GHS receptor in the activated state as well as at lower efficacy, we studied the dynamics of the molecule in the presence of the agonist ghrelin as well as an inverse agonist. By and large, we did not see a conclusive trend of order parameter changes in response to agonist or inverse agonist binding. The bulk measurement as conducted here is likely not sensitive to detect the influence of the few residues that might change their dynamics in response to ligand binding. This suggests that although the energy landscape of the receptor is changed upon ligand binding, the molecular mobility remains on a relatively high level. This agrees with fluorescence studies on the GHS receptor^8^ and the results on the β_2_ adrenergic receptor, which show that the agonist-bound molecule is highly dynamic and interconverts between inactive, intermediate, and active conformations with varying timescales^18^.

### The membrane environment only slightly influences the molecular dynamics of the GHS receptor

Although GPCRs are characteristic for specific tissues and are found in lipid membranes of highly specific compositions, we note that the molecular dynamics of the GHS receptor is also sensitive to the membrane environment. Although unsaturated lipid membranes (such as POPC) are in general more mobile that saturated ones (i.e. DMPC), the molecular mobility of the GHS receptor in DMPC was a bit higher than in POPC^59^. This can be related to the hydrophobic thickness of either membrane, which is higher in POPC than in DMPC, which leads to a slight increase in the α-helix length of the receptor, which rigidifies these residues to decrease the overall order parameters of the molecule^24^,^60^. This underlines the dynamic adaptivity and the structural plasticity of the GHS receptor and possibly of many members of the GPCR family. Such high flexibility increasingly appears as a general property of many GPCRs^23^,^24^ required for the biological function of these molecules.

In summary, although in this preliminary state of the project, no site-specific dynamics information was achieved, the experiments show that the GHS receptor is highly mobile in liquid-crystalline lipid membranes. Given the current resolution of the NMR spectra of the GHS receptor, no site-specific assignments were possible, restricting the conclusions to an average picture, which nevertheless underlines the outstanding position of the physiologically highly important GPCRs among other membrane proteins. In situations, where spectral resolution limits the assignability of the NMR spectra, specific labeling by cell-free synthesis or usage of auxotrophic *E. coli* strains may provide further insights into the molecular dynamics of GPCRs going beyond the initial characterization provided here.

## Methods

### Materials

The lipids 1-palmityl-2-oleyl-*sn*-glycero-3-phospocholine (POPC) or 1,2-dimyristol-*sn*-glycero-3-phosphocholine (DMPC) along with the chain deuterated analog DMPC-*d*_54_ as well as 1,2-dihexanoyl-*sn*-glycero-3-phosphocholine (DHPC) were purchased from Avanti Polar Lipids, Inc. (Alabaster, USA). Chemicals for fermentation were purchased from Sigma-Aldrich (Taufkirchen, Germany) and isotopically labeled ammonium salts and glucose were purchased from Cambridge Isotopes, USA.

### Peptide Synthesis

Neuropeptide Y (NPY), ghrelin and the inverse agonist KbFwLK(Pam)-NH_2_ (b = β-(3-benzothienyl)-D-alanine) were synthesized by Fmoc/*tert*-butyl strategy as described before^28^,^34^. Fluorescent tracer [Dpr^3^-Oct; Dpr^16^-atto520]-ghrelin (Dpr, diaminopropionic acid; Oct, octanoic acid) for *in vitro* functionality assay was prepared similarly as described for a fluorescein-conjugated ghrelin analogue^34^. [Boc-G^1^; Dpr^3^(Mtt); Dpr^16^(Dde)]-ghrelin was synthesized automatically on R-Wang resin. Mtt (4-methyltrityl) protection group was then cleaved by repeated treatment with 94/5/1 dichloromethane/triisopropylsilane/trifluoroacetic acid (v/v/v) (15 × 1 min), and resin was washed with 97.5/2.5 (v/v) dichloromethane/*N,N*-diisopropylethylamine (2 × 10 min). Octanoic acid was coupled using 1-hydroxybenzotriazole/diisopropylcarbodiimide (5 eq. each) in dimethylformamide overnight. For coupling of atto520 dye, Dde (2-acetyldimedone) protection group was cleaved by repeated treatment with 98/2 (v/v) dimethylformamide/hydrazine (10 × 10 min), and 1 eq. of atto520 (free acid; Sigma-Aldrich, Taufkirchen, Germany) was coupled using 1-hydroxybenzotriazole/diisopropycarbodiimide in dimethylformamide overnight. The same protocol was applied for the preparation of [Dpr^22^-atto520]-NPY as control.

### Expression of the GHS receptor

The human wild type GHSR1a including an N-terminal eightfold His-tag was overexpressed in *Escherichia coli* in defined mineral salt medium using a fed-batch fermentation process as described before^42^,^61^. For uniform ^15^N labeling, ^15^NH_4_Cl and (^15^NH_4_)_2_SO_4_ (Cambridge Isotope Lab., USA) was added as sole nitrogen source during fermentation. Furthermore, uniform ^13^C labeling was achieved by applying U-^13^C6 glucose (Cambridge Isotope Lab., USA) during the feed phase 30 minutes prior to the induction of expression through addition of ITPG. Expression times varied from 3 to 4 hours. Preparation of inclusion bodies, followed by the solubilization and the purification of the receptor was conducted as previously recorded^62^.

### Receptor folding and NMR sample preparation

To induce the formation of the disulfide bridge in the GHS receptor, the purified receptor was dialyzed against a folding buffer containing 1 mM oxidized (GSSG) and 2 mM (GSH) reduced glutathione at pH 8, as previously described^24^. For receptor reconstitution POPC or DMPC was suspended in 50 mM NaP (pH 8) reaching a lipid concentration of 10 mg/ml. Subsequently, lipid dispersions were merged with a sixfold (in case of POPC) or fourfold (for DMPC) molar excess of the detergent DHPC by heating to 40 °C. For POPC/DHPC solutions, three cycles of temperature jumps between 0 °C and 40 °C were completed. The receptor dispersion was incubated at a molar ratio of 1:200 (receptor: lipid) and three additional heating/cooling cycles were conducted. Removal of the detergent was accomplished by application of BioBeads SM2 (Bio-Rad Lab., Germany), where 50 mg/ml were added twice. To examine the influence of ligands, after bicelle preparation, either the natural agonist ghrelin or an inverse agonist KbFwLK(Pam)-NH_2_ were added in a molar ratio of 1:2 (receptor:ligand). Preparations were completed by ultracentrifugation of the sample at 86,000 rpm for ~12 hours. Final MAS samples contained ~6 mg of receptor. Samples used for static ^15^N NMR spectra contained ~12 mg of receptor. Chain deuterated DMPC-*d*_54_ instead of DMPC was used to suppress aliphatic lipid signals.

### *In vitro* binding assay

Functionality of GHS receptor preparations was tested by a homogeneous fluorescence binding assay using [Dpr^3^-Oct; Dpr^16^-atto520]-ghrelin (ghrelin-atto520), detecting fluorescence enhancement upon receptor binding^29^. Functionality of the fluorescent tracer ghrelin-atto520 was verified in ^3^H-inositol phosphate accumulation assays as described before displaying an EC_50_ value of 1.7 nM (pEC_50_ = 8.8 ± 0.10; ghrelin: pEC_50_ = 9.0 ± 0.15).

For saturation binding analysis, 25 nM or 100 nM of ghrelin-atto520 was incubated with increasing concentration of GHS receptor containing bicelles in binding buffer (Tris 50 mM pH 7, 10 mM CaCl_2_, 1 mM MgCl_2_, 0.01% TritonX-100) or bicelles prepared in the absence of receptor protein for 2 h under gentle agitation in opaque 96 well plates. For displacement assays, 3.16 μM GHS receptor in buffer was incubated with 100 nM ghrelin-atto 520 and increasing concentrations of unlabeled peptides. Fluorescence intensity was measured in a plate reader (FlexStation 3, Molecular Devices, Sunnyvale, USA); using linear polarized fluorescence light, an excitation wavelength of 520 nm, an emission wavelength of 540 nm (beam splitter 530 nm), PMT medium, 90° detection angle. Experiments were carried out three times independently in triplicates. Data were normalized to fluorescence in unbound state (100%), and combined into a single curve.

Data were fitted to the Hill equation to determine the inflection point. For displacement experiments, Goldstein-Barrett correction^31^ was applied to determine K_i_ values, using the K_D_ of 0.15 nM reported previously^33^.

### NMR experiments

Static ^15^N CP NMR spectra were acquired on a Bruker Avance I 750 MHz NMR spectrometer (Bruker Biospin, Rheinstetten) at a temperature of 37 °C. Standard CP spectra with contact times of either 70 μs, 2 ms, or 8 ms were recorded using Hahn echo detection. TPPM decoupling at a radio field strength of ~62 kHz was applied. Typical 90° pulse lengths were 7 μs and 6 μs on the ^1^H and ^15^N channel, respectively. Spectra line shapes were simulated using MathCad, considering anisotropic and isotropic contributions.

^13^C magic-angle spinning (MAS) NMR experiments were carried out on a Bruker Avance III 600 MHz NMR spectrometer using MAS probes equipped with 3.2 or 4 mm spinning modules at a temperature of 37 °C. The 90° pulses were usually set to 4 μs for both, ^1^H and ^13^C channels. The heteronuclear decoupling sequence Spinal64 was used at a field strength of ~65 kHz. One dimensional ^13^C NMR experiments (either CP MAS, directly excited and INEPT experiments) were conducted at a MAS frequency of 7 kHz. For the ^13^C CP MAS NMR spectra, a contact time of 700 μs was used. The ^1^H-^13^C dipolar couplings were measured using the DipShift pulse sequence^43^ at a MAS frequency of 5 kHz. For homonuclear ^1^H-^1^H decoupling, the FSLG sequence was used^63^. Excitation of ^13^C nuclei was either accomplished by direct excitation or applying CP with contact times ranging from 20 μs to 2 ms. While using a short contact time of 20 μs the RODEO DipShift experiment^64^ was used to avoid distortion effects in the dephasing curves. Dephasing signals were acquired over one rotor period for all resolved isotropic chemical shift signals. Averaged dipolar coupling strengths were determined by best fit of experimental dephasing curves as described in the literature^50^. Order parameters were calculated as the ratio of the motionally averaged dipolar coupling strength and the rigid limit value of the dipolar coupling as determined experimentally^50^,^65^. R-PDLF experiments using a R18^7^_1_^45^ pulse scheme were carried out at 5 kHz MAS frequency. Polarization transfer was accomplished by an INEPT step. For ^1^H and ^13^C pulse lengths of 5 μs were used. The decoupling field strength by use of Spinal 64 was set to 20 kHz to allow acquisition of a larger number of increments in the indirect dimension.

## Additional Information

**How to cite this article**: Schrottke, S. *et al*. Expression, Functional Characterization, and Solid-State NMR Investigation of the G Protein-Coupled GHS Receptor in Bilayer Membranes. *Sci. Rep.* **7**, 46128; doi: 10.1038/srep46128 (2017).

**Publisher's note:** Springer Nature remains neutral with regard to jurisdictional claims in published maps and institutional affiliations.

## Supplementary Material

Supporting Information

## References

[b1] Rosenbaum D. M. . GPCR engineering yields high-resolution structural insights into beta(2)-adrenergic receptor function. Science 318, 1266–1273 (2007). 17962519 10.1126/science.1150609

[b2] Warne T. . Structure of a beta(1)-adrenergic G-protein-coupled receptor. Nature 454, 486–491 (2008). 18594507 10.1038/nature07101 PMC2923055

[b3] Steyaert J. & Kobilka B. K. Nanobody stabilization of G protein-coupled receptor conformational states. Curr. Opin. Struct. Biol. 21, 567–572 (2011). 21782416 10.1016/j.sbi.2011.06.011 PMC3166880

[b4] Venkatakrishnan A. J. . Molecular signatures of G-protein-coupled receptors. Nature. 494, 185–194 (2013). 23407534 10.1038/nature11896

[b5] Frauenfelder H., Sligar S. G. & Wolynes P. G. The energy landscapes and motions of proteins. Science. 254, 1598–1603 (1991). 1749933 10.1126/science.1749933

[b6] Henzler-Wildman K. & Kern D. Dynamic personalities of proteins. Nature. 450, 964–972 (2007). 18075575 10.1038/nature06522

[b7] Huster D. Investigations of the structure and dynamics of membrane-associated peptides by magic angle spinning NMR. Prog. Nucl. Magn. Reson. Spectrosc. 46, 79–107 (2005).

[b8] Mary S. . Ligands and signaling proteins govern the conformational landscape explored by a G protein-coupled receptor. Proc. Natl. Acad. Sci. USA 109, 8304–8309 (2012). 22573814 10.1073/pnas.1119881109 PMC3361445

[b9] Fay J. F. & Farrens D. L. Structural dynamics and energetics underlying allosteric inactivation of the cannabinoid receptor CB1. Proc. Natl. Acad. Sci. USA 112, 8469–8474 (2015). 26100912 10.1073/pnas.1500895112 PMC4500223

[b10] Yao X. . Coupling ligand structure to specific conformational switches in the beta2-adrenoceptor. Nat. Chem. Biol. 2, 417–422 (2006). 16799554 10.1038/nchembio801

[b11] Hubbell W. L., Altenbach C., Hubbell C. M. & Khorana H. G. Rhodopsin structure, dynamics, and activation: a perspective from crystallography, site-directed spin labeling, sulfhydryl reactivity, and disulfide cross-linking. Adv. Protein Chem. 63, 243–290 (2003). 12629973 10.1016/s0065-3233(03)63010-x

[b12] Columbus L. & Hubbell W. L. A new spin on protein dynamics. Trends Biochem. Sci. 27, 288–295 (2002). 12069788 10.1016/s0968-0004(02)02095-9

[b13] Liu J. J. . Biased signaling pathways in beta2-adrenergic receptor characterized by ^19^F NMR. Science 335, 1106–1110 (2012). 22267580 10.1126/science.1215802 PMC3292700

[b14] Okude J. . Identification of a conformational equilibrium that determines the efficacy and functional selectivity of the mu-opioid receptor. Angew. Chem. Int. Ed. 54, 15771–15776 (2015). 10.1002/anie.201508794 PMC4722849 26568421

[b15] Kim T. H. . The role of ligands on the equilibria between functional states of a G protein-coupled receptor. J. Am. Chem. Soc. 135, 9465–9474 (2013). 23721409 10.1021/ja404305k PMC3763947

[b16] Horst R., Liu J. J., Stevens R. C. & Wuthrich K. Beta-adrenergic receptor activation by agonists studied with ^19^F NMR spectroscopy. Angew. Chem. Int. Ed. 52, 10762–10765 (2013). 10.1002/anie.201305286 PMC3925757 23956158

[b17] Kofuku Y. . Efficacy of the beta(2)-adrenergic receptor is determined by conformational equilibrium in the transmembrane region. Nat. Commun. 3, 1045 (2012). 22948827 10.1038/ncomms2046 PMC3658005

[b18] Manglik A. . Structural insights into the dynamic process of beta2-adrenergic receptor signaling. Cell. 161, 1101–1111 (2015). 25981665 10.1016/j.cell.2015.04.043 PMC4441853

[b19] Sounier R. . Propagation of conformational changes during mu-opioid receptor activation. Nature. 524, 375–378 (2015). 26245377 10.1038/nature14680 PMC4820006

[b20] Deupi X. & Kobilka B. K. Energy landscapes as a tool to integrate GPCR structure, dynamics, and function. Physiology. 25, 293–303 (2010). 20940434 10.1152/physiol.00002.2010 PMC3056154

[b21] Manglik A. & Kobilka B. The role of protein dynamics in GPCR function: insights from the beta2AR and rhodopsin. Curr. Opin. Cell Biol. 27, 136–143 (2014). 24534489 10.1016/j.ceb.2014.01.008 PMC3986065

[b22] Park S. H. . Local and global dynamics of the G protein-coupled receptor CXCR1. Biochemistry. 50, 2371–2380 (2011). 21323370 10.1021/bi101568j PMC3236025

[b23] Schmidt P. . The G protein-coupled neuropeptide Y receptor type 2 is highly dynamic in lipid membranes as revealed by solid-state NMR spectroscopy. Chemistry 20, 4986–4992 (2014). 24623336 10.1002/chem.201304928

[b24] Thomas L. . The molecular dynamics of the G protein-coupled neuropeptide Y2 receptor in monounsaturated membranes investigated by solid-state NMR spectroscopy. J. Biomol. NMR 61, 347–359 (2015). 25556885 10.1007/s10858-014-9892-5

[b25] Wiedmer P. . Ghrelin, obesity and diabetes. Nat. Clin. Pract. Endocrinol. Metab. 3, 705–712 (2007). 17893689 10.1038/ncpendmet0625

[b26] Vortmeier G. . Integrating solid-state NMR and computational modeling to investigate the structure and dynamics of membrane-associated ghrelin. PLoS. One. 10, e0122444 (2015). 25803439 10.1371/journal.pone.0122444 PMC4372444

[b27] Kojima M. . Ghrelin is a growth-hormone-releasing acylated peptide from stomach. Nature. 402, 656–660 (1999). 10604470 10.1038/45230

[b28] Kostelnik K. B. . High metabolic in vivo stability and bioavailability of a palmitoylated ghrelin receptor ligand assessed by mass spectrometry. Bioorg. Med. Chem. 23, 3925–3932 (2015). 25541202 10.1016/j.bmc.2014.12.008

[b29] Casiraghi M. . Functional modulation of a G protein-coupled receptor conformational landscape in a lipid bilayer. J. Am. Chem. Soc. 138, 11170–11175 (2016). 27489943 10.1021/jacs.6b04432

[b30] Hulme E. C. & Trevethick M. A. Ligand binding assays at equilibrium: validation and interpretation. Br. J. Pharmacol. 161, 1219–1237 (2010). 20132208 10.1111/j.1476-5381.2009.00604.x PMC3000649

[b31] Goldstein A. & Barrett R. W. Ligand dissociation constants from competition binding assays: errors associated with ligand depletion. Mol. Pharmacol. 31, 603–609 (1987). 3600604

[b32] Carter C. M., Leighton-Davies J. R. & Charlton S. J. Miniaturized receptor binding assays: complications arising from ligand depletion. J. Biomol. Screen. 12, 255–266 (2007). 17259589 10.1177/1087057106297788

[b33] Els S. . An aromatic region to induce a switch between agonism and inverse agonism at the ghrelin receptor. J. Med. Chem. 55, 7437–7449 (2012). 22920150 10.1021/jm300414b

[b34] Park M. . Bioorthogonal Labeling of Ghrelin Receptor to Facilitate Studies of Ligand-Dependent Conformational Dynamics. Chem. Biol. 22, 1431–1436 (2015). 26548612 10.1016/j.chembiol.2015.09.014

[b35] Damian M. . Ghrelin receptor conformational dynamics regulate the transition from a preassembled to an active receptor:Gq complex. Proc. Natl. Acad. Sci. USA 112, 1601–1606 (2015). 25605885 10.1073/pnas.1414618112 PMC4321262

[b36] Opella S. J. Protein dynamics by solid state nuclear magnetic resonance. Methods Enzymol. 131, 327–361 (1986). 3773765 10.1016/0076-6879(86)31048-6

[b37] Reuther G. . The lipidated membrane anchor of the N-ras protein shows an extensive dynamics as revealed by solid-state NMR. J. Am. Chem. Soc. 128, 13840–13846 (2006). 17044712 10.1021/ja063635s

[b38] Good D. B. . Conformational dynamics of a seven transmembrane helical protein Anabaena Sensory Rhodopsin probed by solid-state NMR. J. Am. Chem. Soc. 136, 2833–2842 (2014). 24467417 10.1021/ja411633w

[b39] Yang J., Aslimovska L. & Glaubitz C. Molecular dynamics of proteorhodopsin in lipid bilayers by solid-state NMR. J. Am. Chem. Soc. 133, 4874–4881 (2011). 21401038 10.1021/ja109766n

[b40] Lorieau J. L. & McDermott A. E. Conformational flexibility of a microcrystalline globular protein: Order parameters by solid-state NMR spectroscopy. J. Am. Chem. Soc. 128, 11505–11512 (2006). 16939274 10.1021/ja062443u

[b41] Park S. H. . Structure of the chemokine receptor CXCR1 in phospholipid bilayers. Nature. 491, 779–783 (2012). 23086146 10.1038/nature11580 PMC3700570

[b42] Kaiser A. . Unwinding of the C-Terminal Residues of Neuropeptide Y is critical for Y(2) Receptor Binding and Activation. Angew. Chem. Int. Ed. 54, 7446–7449 (2015). 10.1002/anie.201411688 PMC5497120 25924821

[b43] Munowitz M. G., Griffin R. G., Bodenhausen G. & Huang T. H. Two-dimensional rotational spin-echo nuclear magnetic resonance in solids: correlation of chemical shift and dipolar interactions. J. Am. Chem. Soc. 103, 2529–2533 (1981).

[b44] Waugh J. S. Uncoupling of local field spectra in nuclear magnetic resonance: determination of atomic positions in solids. Proc. Natl. Acad. Sci. USA 73, 1394–1397 (1976). 1064013 10.1073/pnas.73.5.1394 PMC430300

[b45] Dvinskikh S. V., Zimmermann H., Maliniak A. & Sandstrom D. Measurements of motionally averaged heteronuclear dipolar couplings in MAS NMR using R-type recoupling. J. Magn Reson. 168, 194–201 (2004). 15140427 10.1016/j.jmr.2004.03.001

[b46] Damian M. . High constitutive activity is an intrinsic feature of ghrelin receptor protein: a study with a functional monomeric GHS-R1a receptor reconstituted in lipid discs. J. Biol. Chem. 287, 3630–3641 (2012). 22117076 10.1074/jbc.M111.288324 PMC3281683

[b47] Saurel O. . Local and Global Dynamics in Klebsiella pneumoniae Outer Membrane Protein a in Lipid Bilayers Probed at Atomic Resolution. J. Am. Chem. Soc. 139, 1590–1597 (2017). 28059506 10.1021/jacs.6b11565

[b48] Lee S. . Structural dynamics and thermostabilization of neurotensin receptor 1. J. Phys. Chem. B. 119, 4917–4928 (2015). 25807267 10.1021/jp510735f PMC4564841

[b49] Ding X., Zhao X. & Watts A. G-protein-coupled receptor structure, ligand binding and activation as studied by solid-state NMR spectroscopy. Biochem. J. 450, 443–457 (2013). 23445222 10.1042/BJ20121644

[b50] Huster D., Xiao L. & Hong M. Solid-state NMR investigation of the dynamics of soluble and membrane-bound colicin Ia channel-forming domain. Biochemistry 40, 7662–7674 (2001). 11412120 10.1021/bi0027231

[b51] Liang B., Arora A. & Tamm L. K. Fast-time scale dynamics of outer membrane protein A by extended model-free analysis of NMR relaxation data. Biochim. Biophys. Acta. 1798, 68–76 (2010). 19665446 10.1016/j.bbamem.2009.07.022 PMC2812607

[b52] Vogel A., Scheidt H. A. & Huster D. The distribution of lipid attached EPR probes in bilayers. Application to membrane protein topology. Biophys. J. 85, 1691–1701 (2003). 12944284 10.1016/S0006-3495(03)74599-8 PMC1303343

[b53] Quint S. . Residue-specific side-chain packing determines the backbone dynamics of transmembrane model helices. Biophys. J. 99, 2541–2549 (2010). 20959095 10.1016/j.bpj.2010.08.031 PMC2955363

[b54] Yao H. & Hong M. Membrane-dependent conformation, dynamics, and lipid interactions of the fusion peptide of the paramyxovirus PIV5 from solid-state NMR. J. Mol. Biol. 425, 563–576 (2013). 23183373 10.1016/j.jmb.2012.11.027 PMC4082994

[b55] Lu J. X., Yau W. M. & Tycko R. Evidence from solid-state NMR for nonhelical conformations in the transmembrane domain of the amyloid precursor protein. Biophys. J. 100, 711–719 (2011). 21281586 10.1016/j.bpj.2010.12.3696 PMC3030151

[b56] Barré P., Yamaguchi S., Saito H. & Huster D. Backbone dynamics of bacteriorhodopsin as studied by ^13^C solid-state NMR spectroscopy. Eur. Biophys. J. 32, 578–584 (2003). 12830331 10.1007/s00249-003-0305-z

[b57] Saito H. Dynamic pictures of membrane proteins in two-dimensional crystal, lipid bilayer and detergent as revealed by site-directed solid-state C-13 NMR. Chem. Phys. Lipids 132, 101–112 (2004). 15530452 10.1016/j.chemphyslip.2004.09.009

[b58] Yao H. & Hong M. Conformation and lipid interaction of the fusion peptide of the paramyxovirus PIV5 in anionic and negative-curvature membranes from solid-state NMR. J. Am. Chem. Soc. 136, 2611–2624 (2014). 24428385 10.1021/ja4121956 PMC3985871

[b59] Huster D., Arnold K. & Gawrisch K. Influence of docosahexaenoic acid and cholesterol on lateral lipid organization in phospholipid membranes. Biochemistry 37, 17299–17308 (1998). 9860844 10.1021/bi980078g

[b60] Soubias O., Niu S. L., Mitchell D. C. & Gawrisch K. Lipid-rhodopsin hydrophobic mismatch alters rhodopsin helical content. J. Am. Chem. Soc. 130, 12465–12471 (2008). 18712874 10.1021/ja803599x PMC2538621

[b61] Berger C., Montag C., Berndt S. & Huster D. Optimization of Escherichia coli cultivation methods for high yield neuropeptide Y receptor type 2 production. Protein Expr. Purif. 76, 25–35 (2011). 21055472 10.1016/j.pep.2010.10.012

[b62] Schmidt P. . A reconstitution protocol for the *in vitro* folded human G protein-coupled Y2 receptor into lipid environment. Biophys. Chem. 150, 29–36 (2010). 20421142 10.1016/j.bpc.2010.02.019

[b63] Bielecki A., Kolbert A. C. & Levitt M. H. Frequency-switched pulse sequences: homonuclear decoupling and dilute spin NMR in solids. Chem. Phys. Lett. 155, 341–345 (1989).

[b64] Kurz R. . Avoiding bias effects in NMR experiments for heteronuclear dipole-dipole coupling determinations: principles and application to organic semiconductor materials. Chemphyschem 14, 3146–3155 (2013). 23780575 10.1002/cphc.201300255

[b65] Barré P., Zschörnig O., Arnold K. & Huster D. Structural and dynamical changes of the bindin B18 peptide upon binding to lipid membranes. A solid-state NMR study. Biochemistry 42, 8377–8386 (2003). 12846587 10.1021/bi034239e

