# Supplementary material for: Expression, Functional Characterization, and Solid-State NMR Investigation of the G Protein-Coupled GHS Receptor in Bilayer Membranes
Source: Sci Rep. 2017 Apr 7;7:46128. doi: 10.1038/srep46128 (PMC5384189; doi:10.1038/srep46128)
Supplement: Supporting Information [file srep46128-s1.pdf]

Supporting Information

Expression, Functional Characterization, and Solid-State NMR Investigation of the G Protein-Coupled GHS Receptor in Bilayer Membranes

Stefanie Schrottke[1], Anette Kaiser[2], Gerrit Vortmeier[1], Sylvia Els-Heindl[2], Dennis Worm[2], Mathias Bosse[1], Peter Schmidt[1], Holger A. Scheidt[1], Annette G. Beck-Sickinger[2], Daniel Huster[1,*]

[1] Institute of Medical Physics and Biophysics, University of Leipzig, Härtelstr. 16-18, D-04107 Leipzig, Germany

[2] Institute of Biochemistry, University of Leipzig, Brüderstr. 34, D-04103 Leipzig, Germany

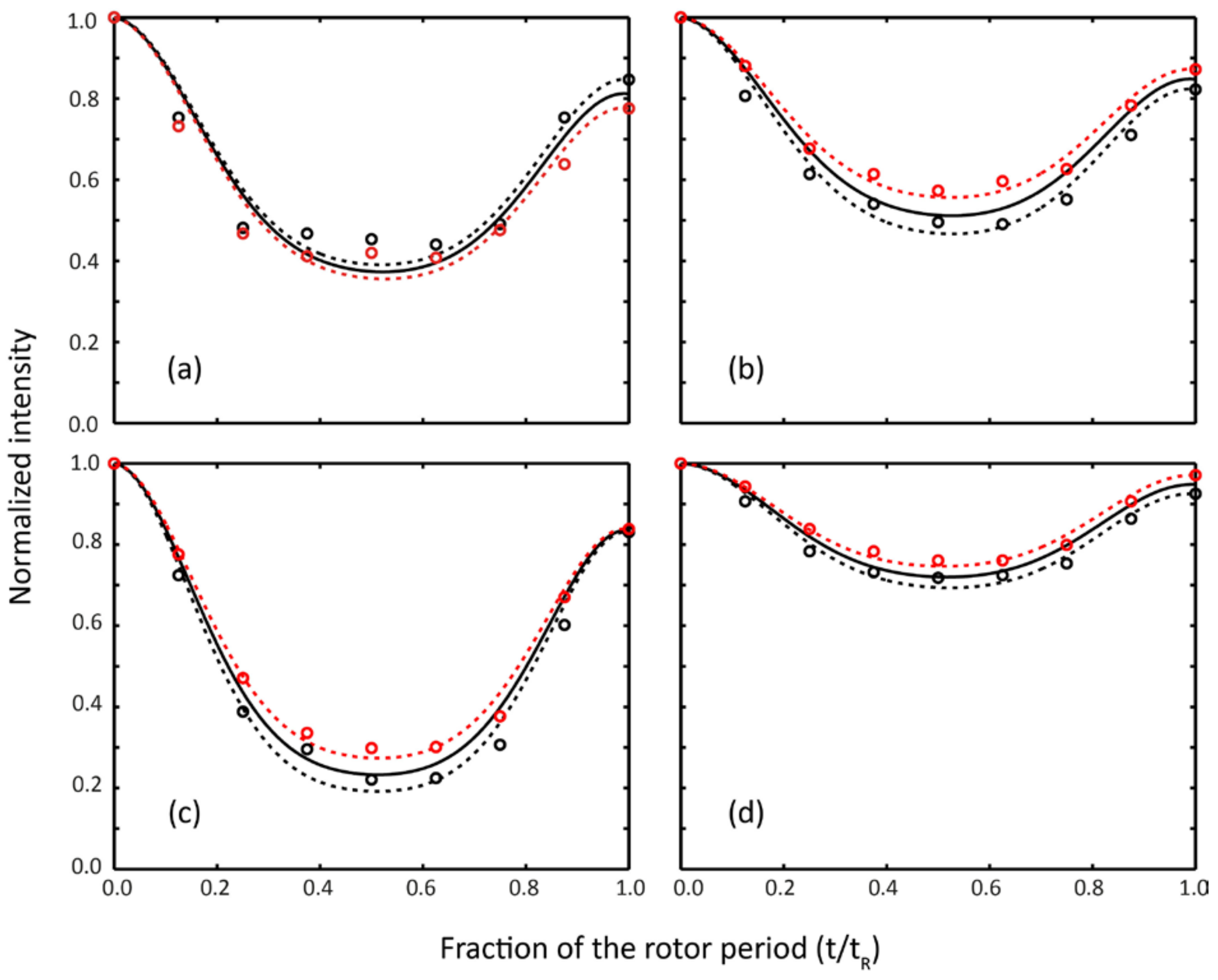

Figure S1. Typical ^{1}H-^{13}C DIPSHIFT dephasing curves acquired for two independent preparations and measurements (black and red) of the GHS receptor reconstituted into DMPC membranes in the absence of any ligand. The individual panels show CH_2 signals (25 ppm) acquired with a CP contact time of 20 μs (a), the same signal with a CP contact time of 700 μs (b), the Cα region acquired with a CP contact time of 700 μs (c), and the CH_3 region also acquired with a CP contact time of 700 μs. The dotted curves show the best fit simulations of the dephasing curves and the solid black curve represents the simulation of the dipolar dephasing assuming the average of the two experimentally determined C-H dipolar coupling values.

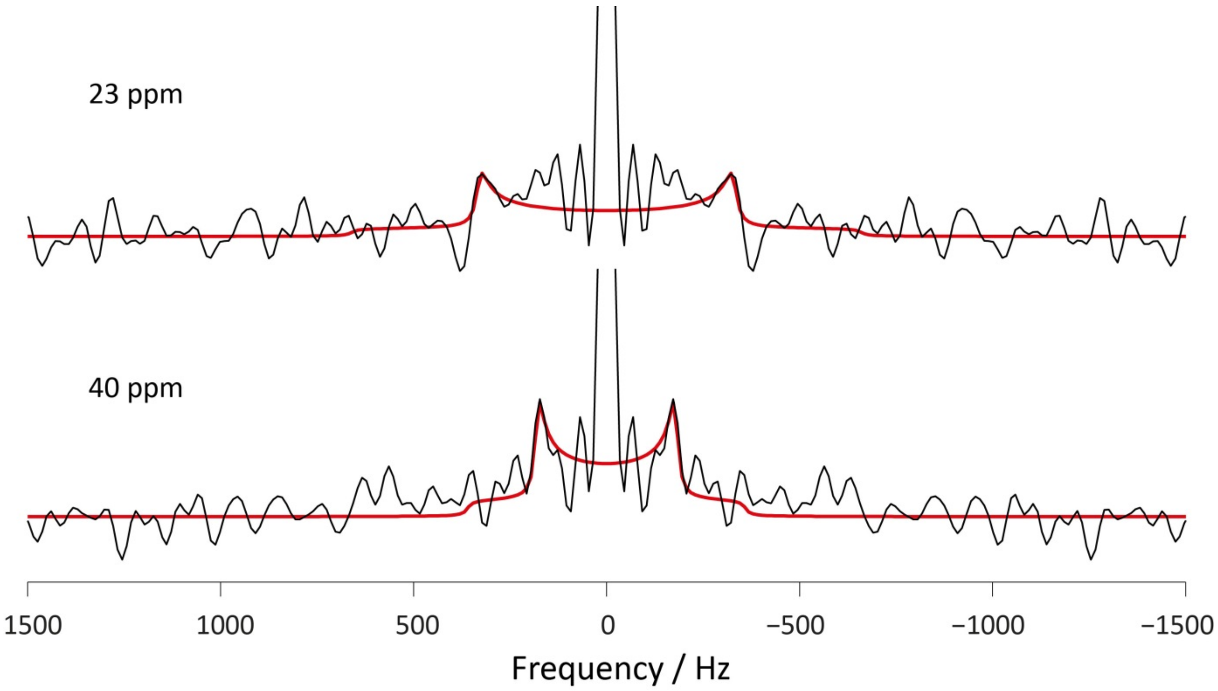

Figure S2. Typical r-PDLF spectra of the GHS receptor in DMPC-d_{54} membranes in the presence of a twofold excess of ghrelin (black lines) and numerical simulations (red lines).

Table S1. DIPSHIFT order parameters of uniformly ^{13}C-labeled GHS receptor reconstituted into DMPC membranes at 37°C and varying CP contact times

	Cα (52-62 ppm)	Gly (43.6 ppm)	CH_2 (40 ppm)	CH_2 (25 ppm)	CH_2 (23 ppm)	CH_3 (15-20 ppm)
w/o ligand						
20 µs CP	0.84 ± 0.02*	0.72 ± 0.03	0.67 ± 0.03	0.52 ± 0.06	0.39 ± 0.02	0.28 ± 0.02
700 µs CP	0.75 ± 0.04	0.64 ± 0.03	0.49 ± 0.03	0.44 ± 0.03	0.31 ± 0.02	0.23 ± 0.01
2 ms CP	0.76 ± 0.03	0.66 ± 0.03	0.46 ± 0.03	0.41 ± 0.02	0.28 ± 0.02	0.21 ± 0.01
direct	0.65 ± 0.03	0.56 ± 0.04	0.41 ± 0.02	0.37 ± 0.01	0.26 ± 0.01	0.20 ± 0.01
w/ ghrelin						
20 µs CP	0.87 ± 0.01	0.76 ± 0.04	0.66 ± 0.04	0.56 ± 0.01	0.44 ± 0.02	0.29 ± 0.01
700 µs CP	0.75 ± 0.02	0.64 ± 0.05	0.50 ± 0.01	0.44 ± 0.02	0.32 ± 0.01	0.23 ± 0.00
2 ms CP	0.76 ± 0.04	0.71 ± 0.05	0.47 ± 0.02	0.45 ± 0.02	0.30 ± 0.01	0.22 ± 0.01
direct	0.65 ± 0.06	0.53 ± 0.05	0.40 ± 0.03	0.38 ± 0.03	0.27 ± 0.01	0.19 ± 0.01
w/ KbFwLK(Pam)-NH_2						
20 µs CP	0.82 ± 0.02	0.76 ± 0.04	0.68 ± 0.00	n.d.**	n.d.	n.d.
700 µs CP	0.76 ± 0.05	0.61 ± 0.04	0.51 ± 0.03	0.45 ± 0.03	0.33 ± 0.05	0.25 ± 0.03
2 ms CP	0.72 ± 0.01	0.61 ± 0.03	0.44 ± 0.01	0.44 ± 0.01	0.30 ± 0.01	0.21 ± 0.01
direct	0.59 ± 0.01	0.51 ± 0.00	0.45 ± 0.05	0.42 ± 0.03	0.28 ± 0.02	0.21 ± 0.02

*Error margins result from two measurements on two independent preparations

** not determined because of insufficient spectral intensity

Table S2. DIPSHIFT order parameters of uniformly ^{13}C-labeled GHS receptor reconstituted into POPC membranes at 37°C and varying CP contact times

	Cα (52-62 ppm)	Gly (43.6 ppm)	CH_2 (40 ppm)	CH_2 (25 ppm)	CH_2 (23 ppm)	CH_3 (15-20 ppm)
w/o ligand						
20 µs CP	0.83 ± 0.04	0.72 ± 0.07	0.69 ± 0.04	0.54 ± 0.03	0.45 ± 0.20	0.29 ± 0.01
700 µs CP	0.76 ± 0.02	0.61 ± 0.07	0.53 ± 0.02	0.44 ± 0.01	0.32 ± 0.20	0.23 ± 0.00
2 ms CP	0.80 ± 0.00	0.67 ± 0.08	0.49 ± 0.02	0.45 ± 0.03	0.29 ± 0.01	0.21 ± 0.01
direct	0.69 ± 0.01	0.60 ± 0.03	0.45 ± 0.01	0.40 ± 0.01	0.27 ± 0.01	0.21 ± 0.01
w/ ghrelin						
20 µs CP	0.94 ± 0.05	0.88 ± 0.04	0.70 ± 0.04	0.45 ± 0.02	0.42 ± 0.20	0.21 ± 0.01
700 µs CP	0.79 ± 0.01	0.67 ± 0.03	0.52 ± 0.00	0.45 ± 0.02	0.35 ± 0.30	0.23 ± 0.00
2 ms CP	0.79 ± 0.00	0.72 ± 0.01	0.52 ± 0.02	0.45 ± 0.03	0.31 ± 0.04	0.23 ± 0.02
direct	0.68 ± 0.02	0.56 ± 0.08	0.47 ± 0.01	0.41 ± 0.01	0.25 ± 0.02	0.21 ± 0.01
w/ KbFwLK(Pam)-NH_2						
20 µs CP	0.87 ± 0.04	0.80 ± 0.04	0.74 ± 0.04	0.58 ± 0.03	0.51 ± 0.30	0.29 ± 0.02
700 µs CP	0.81 ± 0.01	0.68 ± 0.03	0.56 ± 0.02	0.47 ± 0.01	0.33 ± 0.01	0.24 ± 0.01
2 ms CP	0.84 ± 0.02	0.61 ± 0.03	0.55 ± 0.02	0.45 ± 0.01	0.30 ± 0.02	0.22 ± 0.00
direct	0.68 ± 0.00	0.57 ± 0.02	0.47 ± 0.04	0.42 ± 0.01	0.29 ± 0.01	0.20 ± 0.01

*Error margins result from two measurements on two independent preparations
